# Supplementary material for: Predicting memory from the network structure of naturalistic events
Source: Nat Commun. 2022 Jul 22;13:4235. doi: 10.1038/s41467-022-31965-2 (PMC9307577; doi:10.1038/s41467-022-31965-2)
Supplement: Supplementary file 1 — Supplementary Information [file 41467_2022_31965_MOESM1_ESM.pdf]

Predicting memory from the network structure of naturalistic events

Hongmi Lee[1*], Janice Chen[1]
[1]Department of Psychological and Brain Sciences,
Johns Hopkins University,
Baltimore, MD 21218, USA
*Correspondence: hongmi.lee@jhu.edu

Supplementary Methods

Causality judgment instructions.

Your job is to identify and make a list of event pairs that are causally related to each other within each movie. How can we decide whether two events are causally related or not? In an extremely broad sense, one might say that any event that happened before a target event could be at least partially responsible for the event to happen (e.g., you were born because there was Big Bang), but this wouldn't give us very useful information. So we want to identify only those event pairs that are more strongly related, and you will need to use your own best judgment to decide whether the causal relationship is strong enough. For example, if we have a movie like below,

> Event 1: Jane orders a crab cake at a restaurant.
> Event 2: Jane finds a dead fly in her crab cake.
> Event 3: Jane complains to the manager of the restaurant.

You may say that there is a causal relationship between Event 2 and Event 3, but not between Event 1 and Event 3. We don't really have strict rules or criteria, so it is up to your subjective judgment. But please try to keep your criteria as consistent as possible.

Semantic narrative networks based on word-level information.

To test the effects of semantic centrality based on word-level rather than sentence-level similarity between movie event annotations, we created two additional types of semantic narrative networks. First, we created narrative networks whose edge weights between events were defined as Jaccard indices reflecting the word overlap (exact matching words) between

event text descriptions. The Jaccard indices were computed within each annotator, and then averaged across annotators within each movie. As in the USE-based narrative networks, event centrality was defined as the normalized node degree. We found that the semantic centrality computed from the networks based on Jaccard indices was positively correlated with the semantic centrality based on USE embeddings ($r(202) = .64$, $p < .001$, 95% CI = [.55, .71]) and also with recall probability ($r(202) = .27$, $p < .001$, 95% CI = [.14, .39]). Second, we created networks whose edge weights between events were the cosine similarity between the word embeddings of the events. Specifically, the word embedding of an event was generated by averaging the word vectors (based on Google's pre-trained Word2Vec model; GoogleNews-vectors-negative300-SLIM) of unique words contained in the text description of the event, separately for each annotator. The word embeddings were then averaged across annotators. Words that were not included in the pre-trained Google database were excluded from the analysis. The centrality (normalized node degree) computed from these networks was again positively correlated with the USE-based semantic centrality ($r(202) = .54$, $p < .001$, 95% CI = [.43, .63]) and with recall probability ($r(202) = .34$, $p < .001$, 95% CI = [.21, .46]).

Supplementary Table 1. Movie stimuli details.

Order	Title (*animation)	Duration (min:sec)	Original title	Release year	Director(s)
1	Catch Me If You Can	5:46	Catch Me If You Can	2002	Steven Spielberg
2	The Record*	2:12	A Single Life	2014	Marieke Blaauw, Joris Oprins, Job Roggeveen
3	The Boyfriend	7:45	High Maintenance	2006	Phillip Van
4	The Shoe	2:09	How They Get There	1997	Spike Jonze
5	Keith Reynolds*	5:48	Keith Reynolds Can't Make It Tonight	2008	Felix Massie
6	The Rock*	5:25	An Object at Rest	2015	Seth Boyden
7	The Prisoner	4:20	Arrival (First episode of the TV series "The Prisoner")	1967	Don Chaffey
8	The Black Hole	2:22	The Black Hole	2008	Philip Sansom, Olly Williams
9	Post-It Love	2:41	Post-It Love	2009	Simon Atkinson, Adam Townley
10	Bus Stop	6:54	Stray Dogs	2015	Minka Farthing-Kohl

Supplementary Table 2. Descriptive statistics for movie annotations (title scenes excluded).

Movie	Number of events	Mean event dur. (sec)	Mean number of words used to describe each event				Mean number of sub-events within each event			
			RC	JL	KM	Mean	RC	JL	KM	Mean
Catch Me If You Can	23	15.1	50.3	52.7	71.8	58.3	2.6	2.5	2.5	2.5
The Record	14	9.4	37.3	65.7	78.4	60.5	2.1	3.5	2.8	2.8
The Boyfriend	25	18.4	48.3	71.4	89.1	69.6	2.9	4.1	3.4	3.5
The Shoe	12	10.8	66.2	46.4	49.7	54.1	3.2	2.4	2.2	2.6
Keith Reynolds	25	14.1	30	61.2	70.4	53.9	2.0	2.3	1.7	2
The Rock	27	12.0	35.6	53.4	44.8	44.6	1.9	3.2	1.9	2.3
The Prisoner	16	16.3	53.4	66.4	97.8	72.5	2.8	3.1	3.2	3.0
The Black Hole	10	14.3	88.3	67.1	87	80.8	3.6	4.2	3.2	3.7
Post-It Love	15	10.7	31.6	42.5	41.5	38.5	1.7	2.3	1.7	1.9
Bus Stop	35	11.9	30.4	59.7	48.5	46.2	1.6	2.7	1.9	2.1
Mean across movies	20.2	13.3	47.1	58.7	67.9	57.9	2.4	3.0	2.5	2.6

Supplementary Table 3. An example movie annotation of “The Record” with event-wise ranks based on semantic centrality and causal centrality (annotation by the annotator RC).

Semantic centrality rank	Causal centrality rank	Event description
14	10	The camera pans into an animated scene with a teenage girl in a room in a tall building. The camera is inside the apartment, and the girl opens the pizza box and grabs a pizza.
13	9	The girl, before she can eat, hears a knock on the door. The girl opens the door and looks around to see no one is there.
9	7	The girl looks down and sees a package for her in an envelope at her doorstep. The girl goes back to her chair and to her pizza and opens the envelope. The girl pulls out a package with a disc inside it that says "A Single Life."
12	7	The girl pulls out the record disc from the package and the title "A film by: Job, Joris, and Marieke"
4	6	The girl gets up and puts the record disc into the record player and puts down the needle. The song on the disc plays and she sits down and begins to eat her pizza.
6	5	The girl is about to eat the pizza but then there is a flash. She stops and then the song plays its lyrics on the song. Part of the pizza is gone.
3	7	She notices the pizza is eaten and then looks at the disc. The camera pans to the disc playing on the record player, and the disc is spinning on the player.
2	1	The girl stops the disc and there's a record scratch. The girl pulls the disc back and forth on the player and pizza disappears and reappears as she tests it back and forth. The girl pulls the disc forth and the pizza pie disappears completely in the box as well as in her hand. Then she pulls it back and the pizza reappears.
1	6	The girl realizes her power and gets up then lifts the needle on the record player. The disc plays the song and then the flash goes to her as a pregnant woman. The woman stops the record player and stops the song from playing.
10	4	The woman pulls the disc forward and back and sees the baby develop and devolve like the pizza before. The woman pulls the disc forward and the baby develops and pops into her arms but the baby starts crying.
5	4	The woman then flashes into her childhood self and looks at herself. The girl goes up to record player and tries to stop it but pops off the needle.
7	3	The girl flashes to her in a wheelchair as an elderly woman and she looks at herself. The woman rolls up her wheelchair but she flashes back to the same scene over and over again, getting frustrated. The woman rolls up again and flashes but then stops rolling up and finds that nothing happens. She then tries to roll really fast but falls back.
8	2	The woman gets up and she is an old woman who needs a walker and is wearing glasses. The woman sees that the song is about to end and tries to get to the record player.
11	8	The woman turns into ashes in a pot in the same nursery home and the record player stops with the needle lifting.

Supplementary Table 4. List of Schaefer 400 parcels used to create regions of interest.

Region of interest	Hemisphere	Schaefer parcel ID	Schaefer parcel name
Posterior medial cortex	Left	154	17Networks_LH_DefaultA_pCunPCC_1
		155	17Networks_LH_DefaultA_pCunPCC_2
		156	17Networks_LH_DefaultA_pCunPCC_3
		157	17Networks_LH_DefaultA_pCunPCC_4
		158	17Networks_LH_DefaultA_pCunPCC_5
		159	17Networks_LH_DefaultA_pCunPCC_6
		160	17Networks_LH_DefaultA_pCunPCC_7
	Right	363	17Networks_RH_DefaultA_pCunPCC_1
		364	17Networks_RH_DefaultA_pCunPCC_2
		365	17Networks_RH_DefaultA_pCunPCC_3
		366	17Networks_RH_DefaultA_pCunPCC_4
		367	17Networks_RH_DefaultA_pCunPCC_5
Early visual cortex	Left	7	17Networks_LH_VisCent_Striate_1
		18	17Networks_LH_VisPeri_StriCal_1
		19	17Networks_LH_VisPeri_StriCal_2
		20	17Networks_LH_VisPeri_ExStrSup_1
	Right	207	17Networks_RH_VisCent_Striate_1
		218	17Networks_RH_VisPeri_StriCal_1
		219	17Networks_RH_VisPeri_StriCal_2

Supplementary Table 5. Relationship between semantic centrality and hippocampal-cortical intersubject functional connectivity (ISFC) during movie watching.

Minimum event duration threshold (sec)	Number of events	Correlation between semantic centrality and hippocampus-PMC ISFC (*a*)			Correlation between semantic centrality and hippocampus-EVC ISFC (*b*)			95% CI[3] of (*a*) - (*b*)
		r	*p*[1]	95% CI[2]	*r*	*p*[1]	95% CI[2]	
27	14	.61	.02	[.11, .86]	-.33	.24	[-.73, .24]	[.1, 1.46]
25.5	16	.59	.02	[.14, .84]	-.32	.23	[-.7, .21]	[.12, 1.42]
24	19	.52	.02	[.09, .79]	-.17	.49	[-.58, .31]	[.01. 1.21]
22.5	26	.49	.01	[.13, .74]	.01	.95	[-.38, .4]	[.04, .87]
21	31	.38	.04	[.02, .64]	.02	.93	[-.34, .37]	[-.04, .72]
19.5	44	.29	.06	[-.01, .54]	.05	.75	[-.25, .34]	[-.07, .53]
18	55	.21	.12	[-.05, .45]	-.02	.88	[-.28, .25]	[-.08, .52]

[1] Uncorrected significance of the Pearson correlation coefficient *r* (two-tailed)
[2] Confidence Interval of the correlation coefficient, [lower bound, upper bound].
[3] Confidence interval of the difference between two overlapping correlations based on dependent groups, computed using the method described in ref.[1].

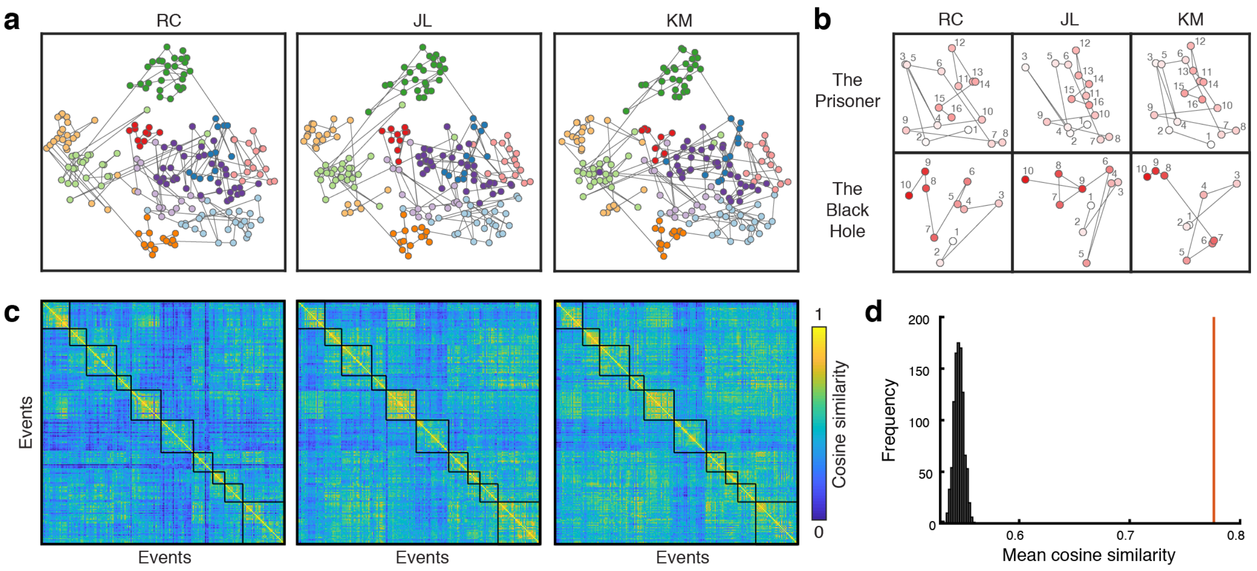

Supplementary Figure 1. Similarity of movie event descriptions across annotators. a. Visualization of three independent annotators' movie event descriptions as trajectories in the Universal Sentence Encoder (USE)[2] text embedding space (for a related method, see ref.[3]). T-distributed stochastic neighbor embedding (t-SNE) was applied on the USE vectors (concatenated across annotators) for dimensionality reduction into a two-dimensional space. Events within each movie formed visible clusters in the space, and the overall configuration of the trajectories was highly similar across annotators. Each dot represents a movie event. Temporally adjacent events are connected with gray lines. Different colors indicate different movies. **b.** Two example movies' annotation trajectories from the three annotators (isolated from the trajectories in **a**). Numbers and the color of dots indicate the order of events within each movie. Dots (events) in brighter colors were presented earlier in the movie. **c.** Cosine similarity between the USE vectors of all 202 events (combined across 10 movies) generated from each annotator's movie event descriptions. Each black square on the diagonal indicates an individual movie (i.e., within-movie similarities). **d.** We performed a randomization test (1000 iterations, one-tailed) to test the statistical significance of the cross-annotator similarity between movie event USE vectors. The red line shows the true mean event-wise cross-annotator cosine similarity between all possible annotator pairs. The histogram shows the null distribution of the mean cross-annotator similarity, generated by shuffling the event labels within each movie and annotator. The mean cross-annotator similarity was significantly greater than zero (M = .78, p = .000999).

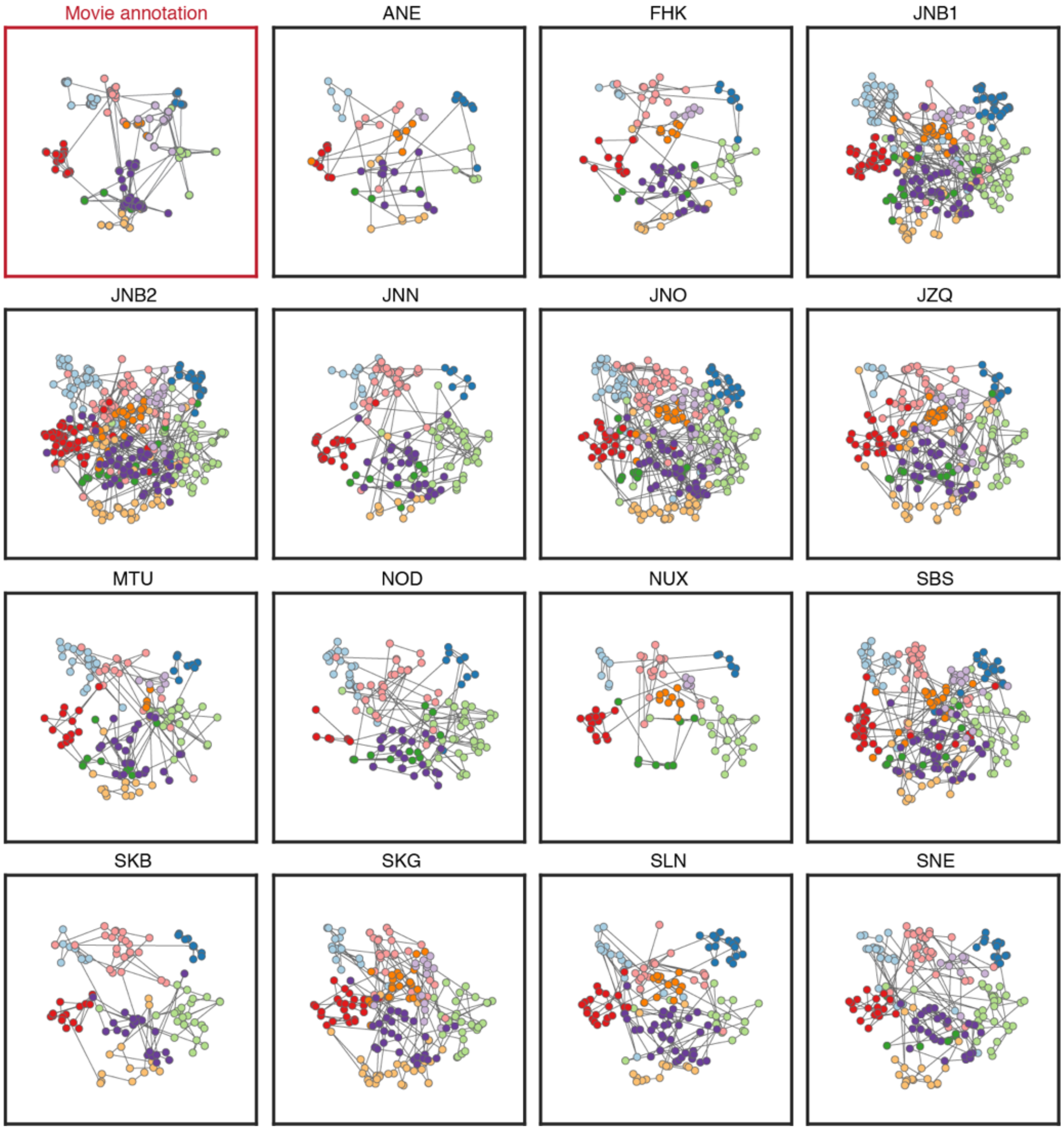

Supplementary Figure 2. Individual participants' recall trajectories in a text embedding space. Each participant's recall transcript was segmented into utterances based on pauses and changes in the topic. Each utterance was transformed into vectors using the Universal Sentence Encoder (USE)[2]. T-distributed stochastic neighbor embedding (t-SNE) was applied on the USE vectors concatenated across all participants' recall transcripts and the movie annotation vectors (averaged across annotators). This allowed us to visualize the USE vectors of the movie annotation (top left cell in the red frame) and recall transcripts (all the other cells in black frames) into a shared two-dimensional space. Each dot in the movie annotation trajectory represents a movie event. Each dot in the recall trajectories represents an utterance during recall. Temporally adjacent events/utterances are connected with gray lines. Different colors indicate different movies. Consistent with a prior study[3], the overall configuration of the recall trajectories was similar to that of the movie annotation trajectory. The recall trajectories were also similar across participants, although the number of movies recalled and the number of utterances made varied across participants.

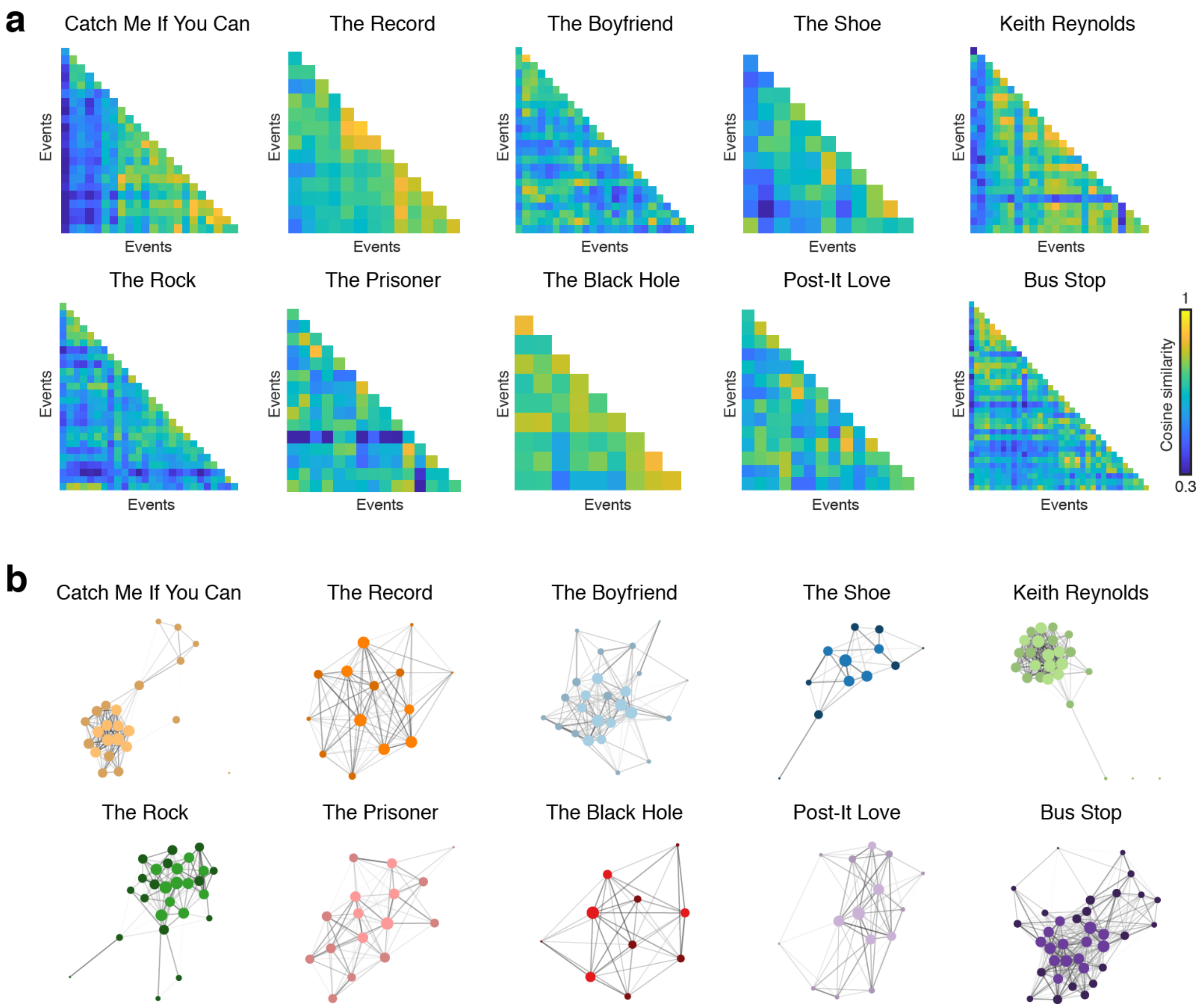

Supplementary Figure 3. Semantic narrative networks of all movie stimuli. a. Semantic similarity matrices of the 10 movies used in the fMRI experiment. **b.** Semantic narrative networks of the 10 movies used in the fMRI experiment. Node size is proportional to centrality (normalized degree) computed from unthresholded networks. Edge thickness is proportional to edge weights. Nodes with brighter colors indicate high (i.e., within the top 40% in each movie) semantic centrality events.

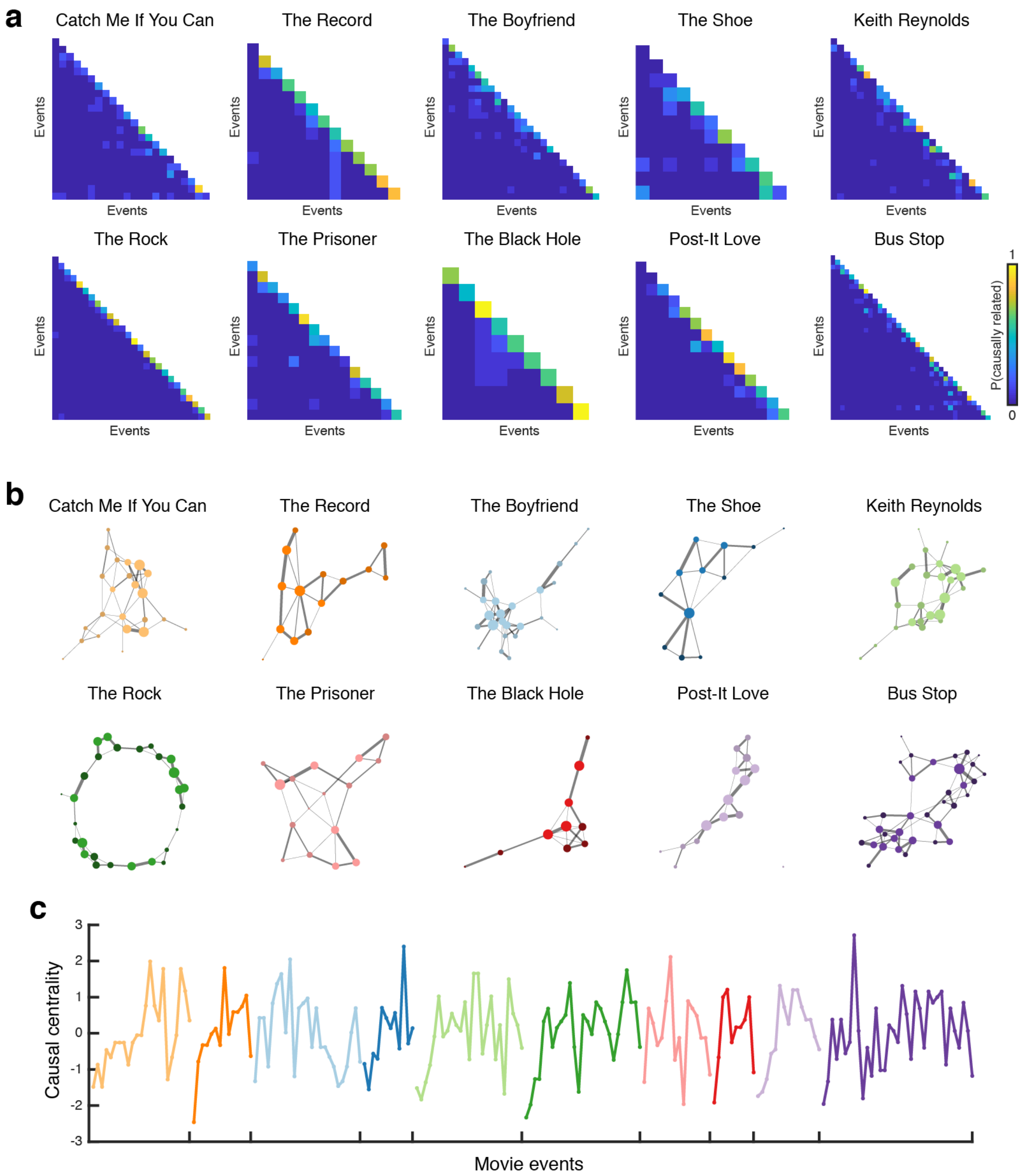

Supplementary Figure 4. Causal narrative networks. a. Causal relationship matrices of the 10 movies used in the fMRI experiment. Causal relatedness between a pair of events within a movie was computed as the proportion of independent coders who identified the pair as causally related. **b.** Causal narrative networks whose nodes are movie events and edge weights are the causal relatedness shown in **a**. Node size is proportional to centrality (normalized degree) computed from unthresholded networks. Edge thickness is proportional to edge weights. Nodes with brighter colors indicate high (i.e., within the top 40% in each movie) causal centrality events. **c.** Causal centrality for individual movie events concatenated across the 10 movies. Different colors denote different movies. Source data are provided as a Source Data file.

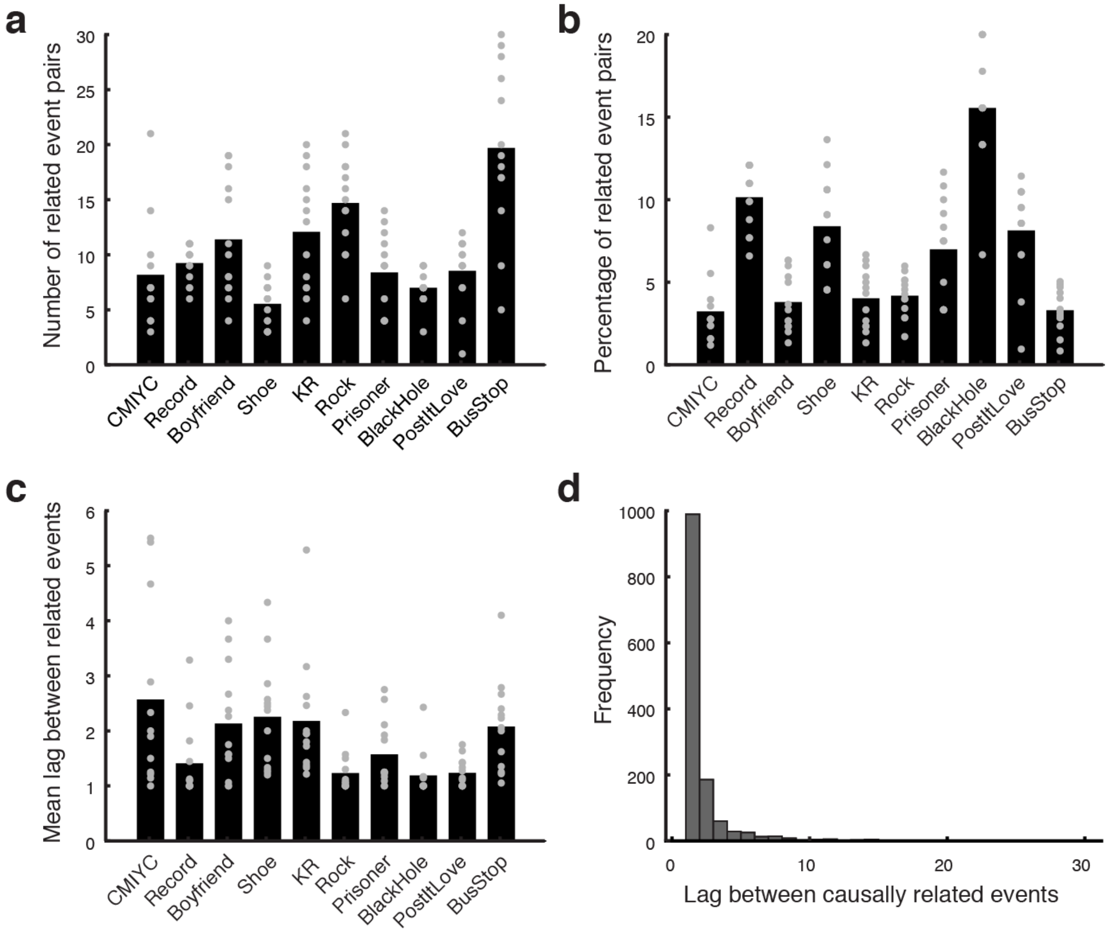

Supplementary Figure 5. Causality rating responses. a. Average number of event pairs identified as causally related within each of the 10 movies used in the fMRI experiment (mean across movies 10.47, s.d. 4.18). **b.** Average percentage of event pairs identified as causally related among all possible event pairs within each movie (mean 6.78 %, s.d. 3.95 %). **c.** Average distance between a pair of causally related events (i.e., the number of events between the two events) within each movie. Lag = 1 if the events are adjacent to each other (mean 1.79 events, s.d. .51 events). **d.** The distribution of lags between causally related events, combined across all movies and coders. Most (73.1%) identified causal relationships occurred between temporally adjacent events. In **a**, **b**, and **c**, gray dots represent individual coders (*N* = 12 for CMIYC, 13 for all other movies) and black bars show the mean across coders. CMIYC = Catch Me If You Can, KR = Keith Reynolds. Source data for **a** – **c** are provided as a Source Data file.

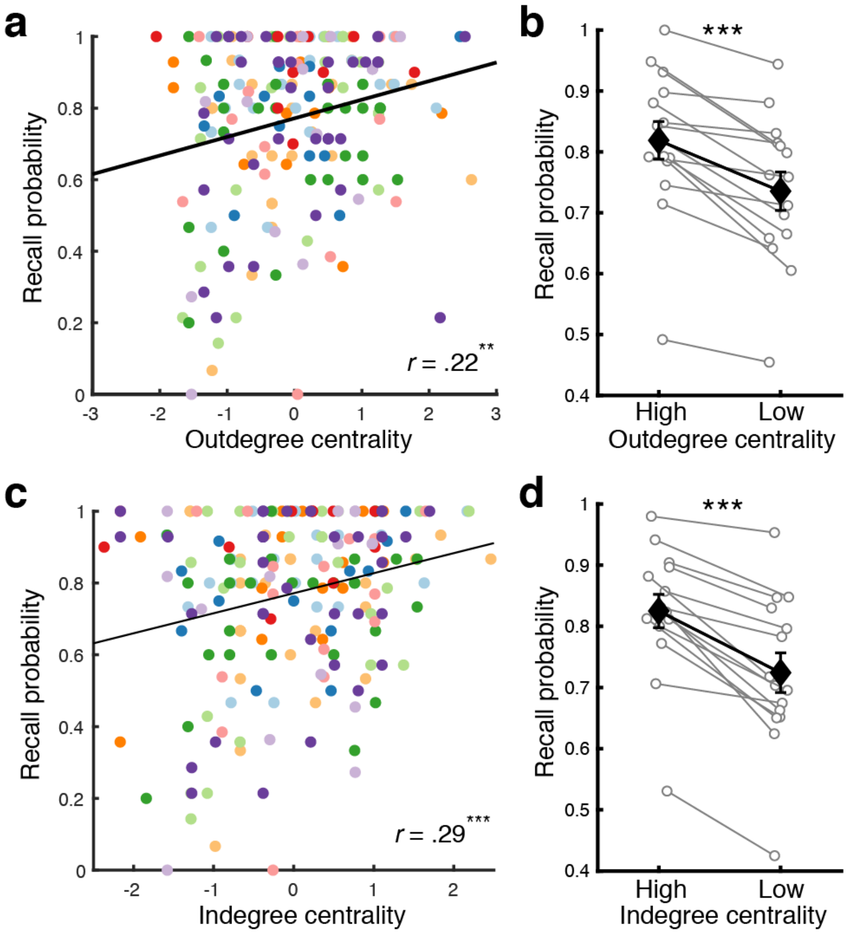

Supplementary Figure 6. Relationship between recall performance and causal centrality computed from directed networks. We generated directed causal narrative networks where source nodes were "cause" events and target nodes were "effect" events. The edge weight of a cause-effect event pair was defined as the proportion of coders who identified the pair as causally related. An event has high outdegree centrality if the event causes many other events. An event has high indegree centrality if the event is caused by many other events. Both outdegree and indegree centrality were positively correlated with the centrality computed from undirected casual narrative networks (outdegree: $r(202) = .79$, $p < .001$, 95% CI = [.73, .83]; indegree: $r(202) = .71$, $p < .001$, 95% CI = [.63, .77]). **a**. Correlation between outdegree centrality and recall probability. **b**. Recall probability for High (top 40%) vs. Low (bottom 40%) outdegree centrality events defined within each movie (averaged across movies). **c**. Correlation between indegree centrality and recall probability. **d**. Recall probability for High (top 40%) vs. Low (bottom 40%) indegree centrality events defined within each movie (averaged across movies). In **a** and **c**, each dot represents an individual movie event. Different colors denote different movies. In **b** and **d**, white circles represent individual participants ($N = 15$). Black diamonds represent the mean across participants within each condition. Error bars show SEM across participants. Two-tailed paired *t*-tests indicated that both higher outdegree ($t(14) = 5.9$, $p = .00004$, Cohen's $d_z = 1.52$, 95% CI of the difference = [.05, .11]) and indegree centrality ($t(14) = 7.34$, $p = .000004$, Cohen's $d_z = 1.9$, 95% CI of the difference = [.07, .13]) were associated with higher recall probability. $^{**}p < .01$, $^{***}p < .001$. Source data are provided as a Source Data file.

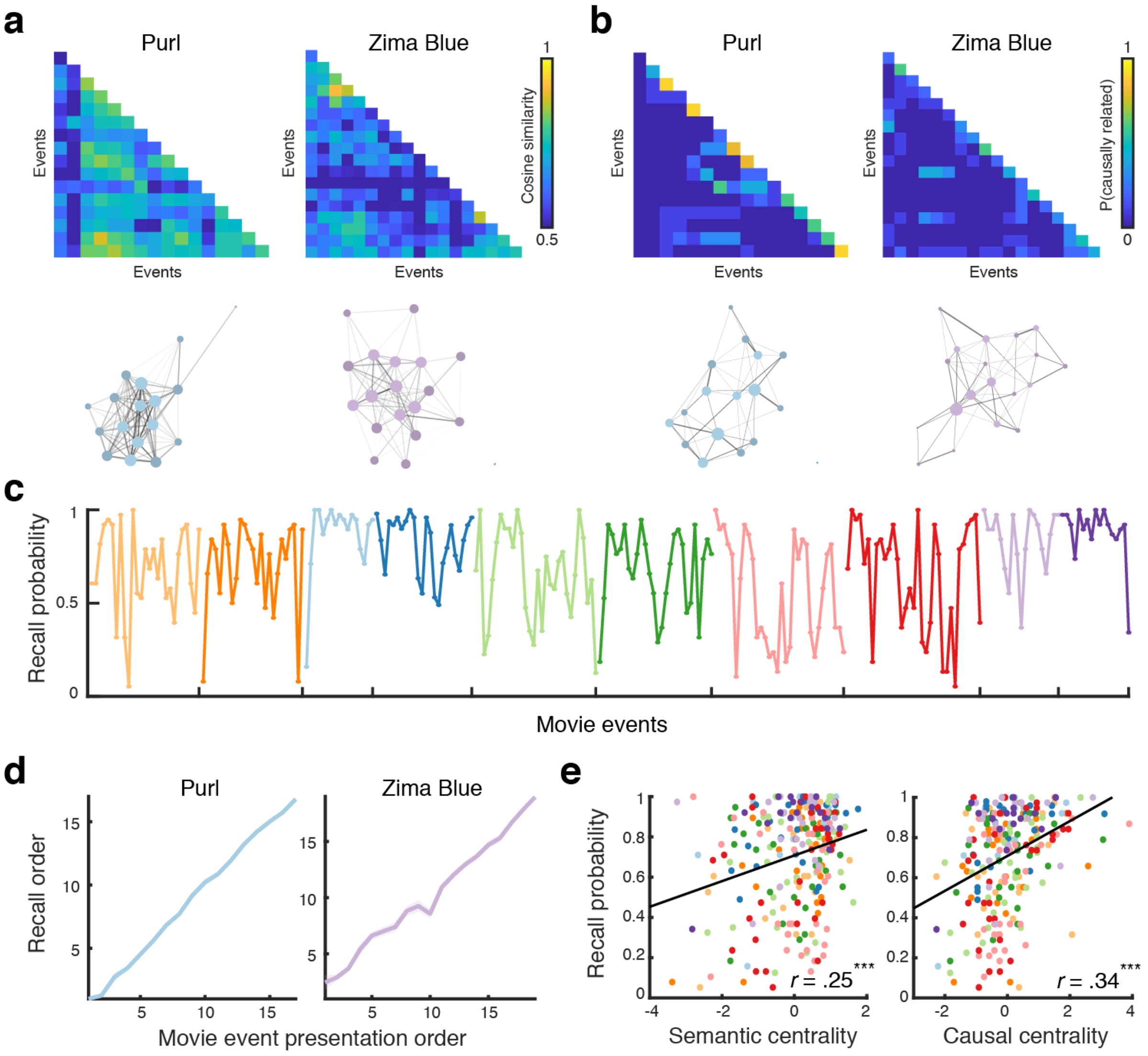

Supplementary Figure 7. Online behavioral experiment. a. The semantic similarity matrices (top) and semantic narrative networks (bottom) of two example movies used in the preregistered online behavioral experiment. **b.** The causal relationship matrices (top) and causal narrative networks (bottom) of the same two example movies shown in **a**. **c.** Recall probability for individual movie events of the ten movies used in the online behavioral experiment, concatenated across movies. As in the fMRI experiment, primacy/recency effects were not observed. Different colors indicate different movies. **d.** Recall order of individual movie events in two example movies. Recall order was calculated as the rank (1 = recalled first, *N* = recalled last, where *N* is the total number of events in the movie) among recalled events. Participants' written recall strictly followed the original event presentation order. **e.** Recall probability was positively correlated with semantic centrality (left; $r(252) = .25$, $p = .00007$, 95% CI = [.13, .36]) and causal centrality (right; $r(252) = .34$, $p < .000001$, 95% CI = [.22, .44]). Each dot represents a movie event. Different colors denote different movies. ***$p < .001$. Source data for **c** – **e** are provided as a Source Data file.

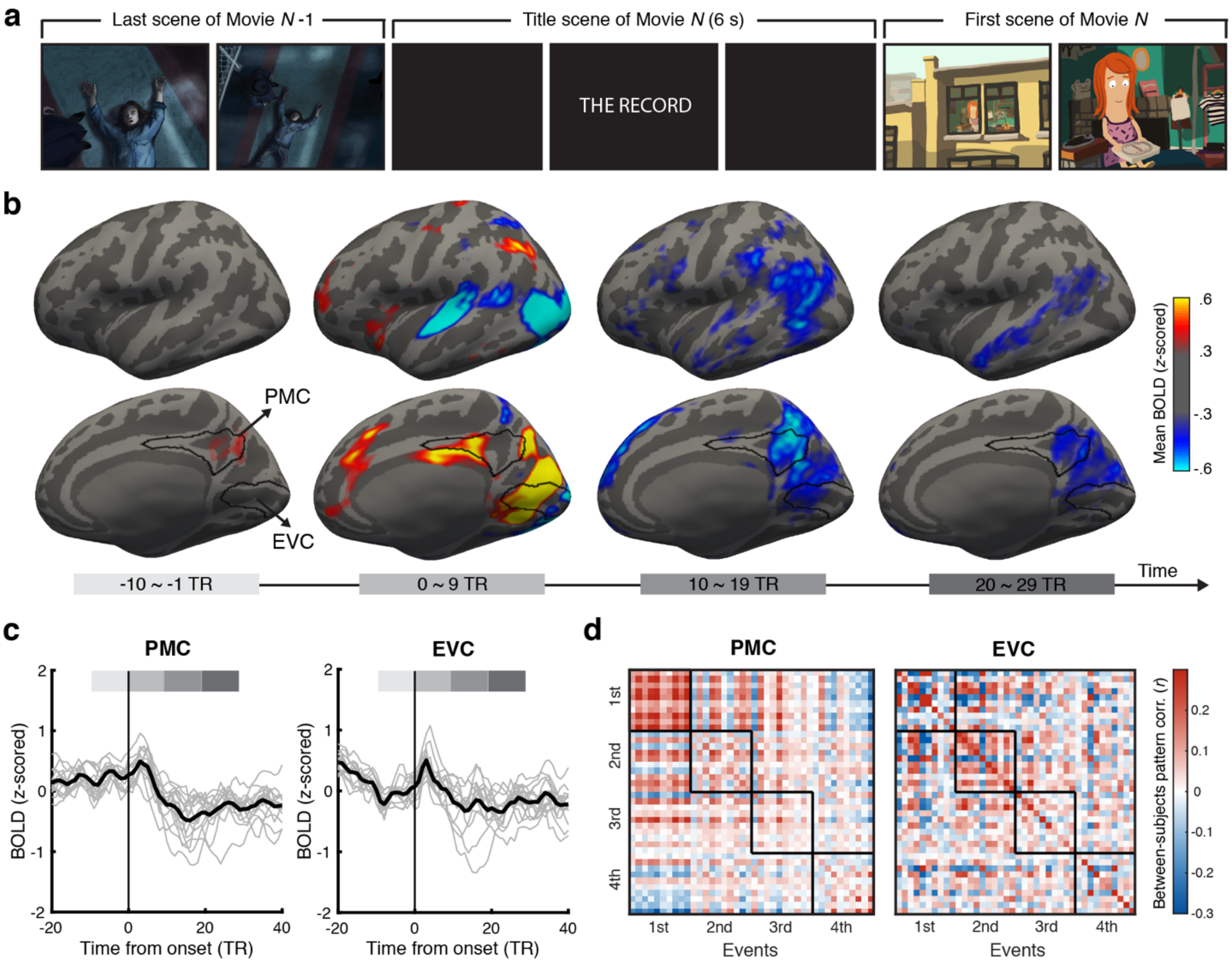

Supplementary Figure 8. Cortical responses at between-movie boundaries during movie watching. a. Example movie frame images around a boundary between two movies presented in the movie watching phase of the fMRI experiment. At between-movie boundaries, the last scene of the preceding movie was followed by a 6-second-long title scene of the upcoming movie. The transition between the 39-s introductory cartoon (presented at the beginning of each scanning run) and the first movie of each scanning run was also counted as a between-movie boundary. **b.** Whole-brain maps of *z*-scored cortical blood oxygenation level dependent (BOLD) signals from 10 TRs before to 29 TRs after between-movie boundaries during movie watching (TR = 1.5 s). The BOLD signals were averaged across times within each 10-TR time window and then across movies and participants. Time zero means the onset of the movie title scene. The maps were arbitrarily thresholded to visualize brain areas whose activation was relatively higher (red-yellow) or lower (cyan-blue) than the mean activation across all time points within a scanning run (z = 0). Between-movie boundaries evoked transient changes in activation across widespread cortical areas. The black outlines indicate the posterior medial cortex (PMC) and early visual cortex (EVC) regions-of-interest. **c.** Activation time courses around between-movie boundaries in PMC (left) and EVC (right). Gray lines show individual participants' time courses, averaged across all between-movie boundaries. Black lines show the averages across participants. The four shades of the gray bars at the top of each panel correspond to the four time windows used in **b**. Source data are provided as a Source Data file. **d.** Intersubject pattern correlation between the mean

activation patterns of the first four events in each of the 10 movies. Each row and column of the similarity matrix represents an event, and the events are grouped by their temporal positions in the movie (i.e., row/column 1 – 10 = the first events of the 10 movies, row/column 11 – 20 = the second events, etc.). The black squares on the diagonal indicate cross-movie similarity within the first, second, third, and fourth events of the movies. In PMC (left), all first events showed similar patterns regardless of specific movies, and this tendency decreased in later events further away from between-movie boundaries. EVC (right) showed relatively weaker pattern similarity across movies within the first events compared to PMC. Movie scene images in **a** were created by the author H. L. using Adobe Illustrator and Adobe Photoshop (adobe.com).

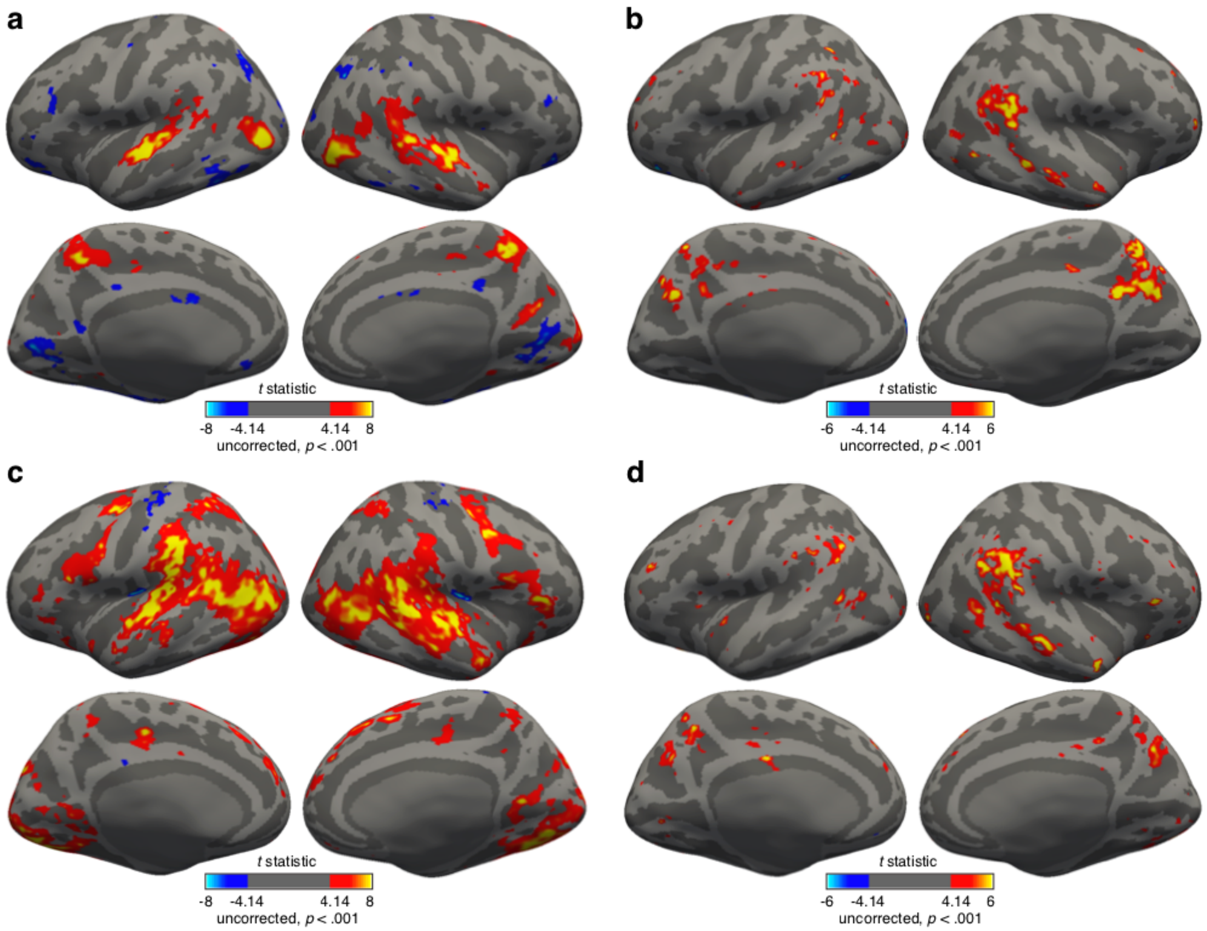

Supplementary Figure 9. Univariate activation. a & b. Whole-brain *t*-statistic maps showing the brain regions whose activation scale with semantic centrality during movie watching (**a**) and recall (**b**). **c & d.** Whole-brain *t*-statistic maps showing the brain regions whose activation scale with causal centrality during movie watching (**c**) and recall (**d**). All maps were liberally thresholded at $p < .001$ (Two-tailed one-sample *t*-tests against zero, uncorrected).

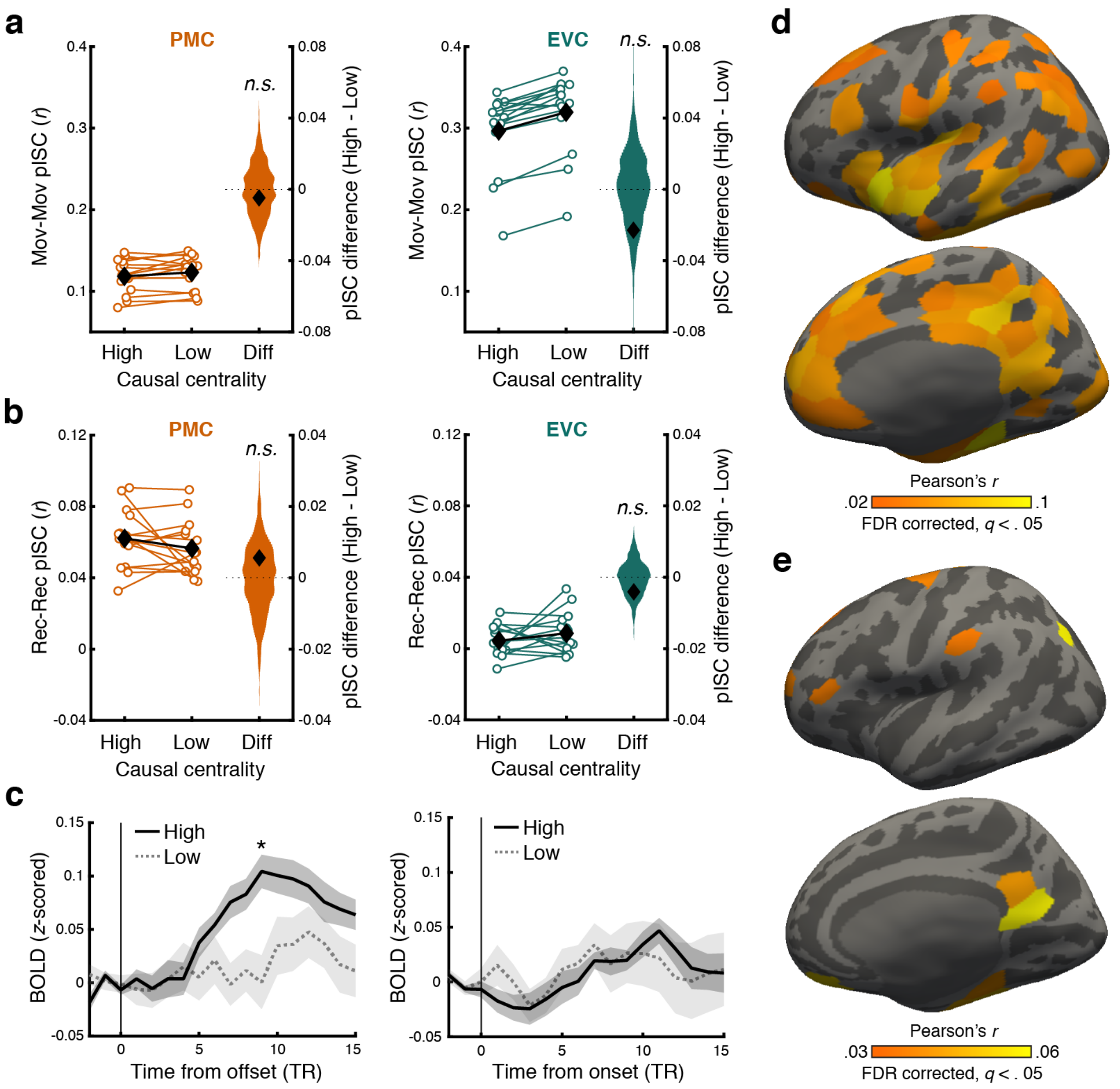

Supplementary Figure 10. Effects of causal narrative structure on neural responses. a & b. Intersubject pattern correlation (pISC) for High vs. Low causal centrality events and the difference (Diff) between the two conditions during movie watching (**a**) and recall (**b**) in the posterior medial cortex (PMC; left panels) and early visual cortex (EVC; right panels). For High and Low causal centrality conditions, white circles represent individual participants (*N* = 15). Black diamonds represent the mean across participants within each condition. Error bars show SEM across participants. For the difference between High and Low conditions (Diff), black diamonds show the true participant average, and histograms show the null distribution of the mean difference. Randomization tests showed that the difference between High vs. Low causal centrality conditions was not significantly different from zero in any of the experimental phases and ROIs (*p*s > .05, two-tailed). **c.** Mean hippocampal blood oxygenation level dependent (BOLD) response time courses aligned at the offset (left) or onset (right) of events during movie watching. Solid lines and dotted lines show responses for the high and low causal centrality events, respectively. Shaded areas indicate SEM across participants. Statistical significance reflects the difference between

High vs. Low centrality events at each time point (two-tailed paired *t*-tests). Higher hippocampal responses were observed following the offset, but not onset, of high causal centrality events. *$q < .05$ (FDR corrected across time points). TR = 1.5 s. **d & e.** Whole-brain representational similarity analysis maps showing the brain regions whose activation patterns reflect the whole causal narrative network structure during movie watching (**d**) and recall (**e**). For each cortical parcel, the causal relationship matrix (Supplementary Figure 3a) of a movie was correlated with the movie's cross-event intersubject fMRI pattern similarity matrix. The correlation coefficients were averaged across movies and participants and then tested for statistical significance against zero using a randomization test (one-tailed). All maps were thresholded at $q < .05$ (FDR-corrected across parcels). Source data for **a** – **c** are provided as a Source Data file.

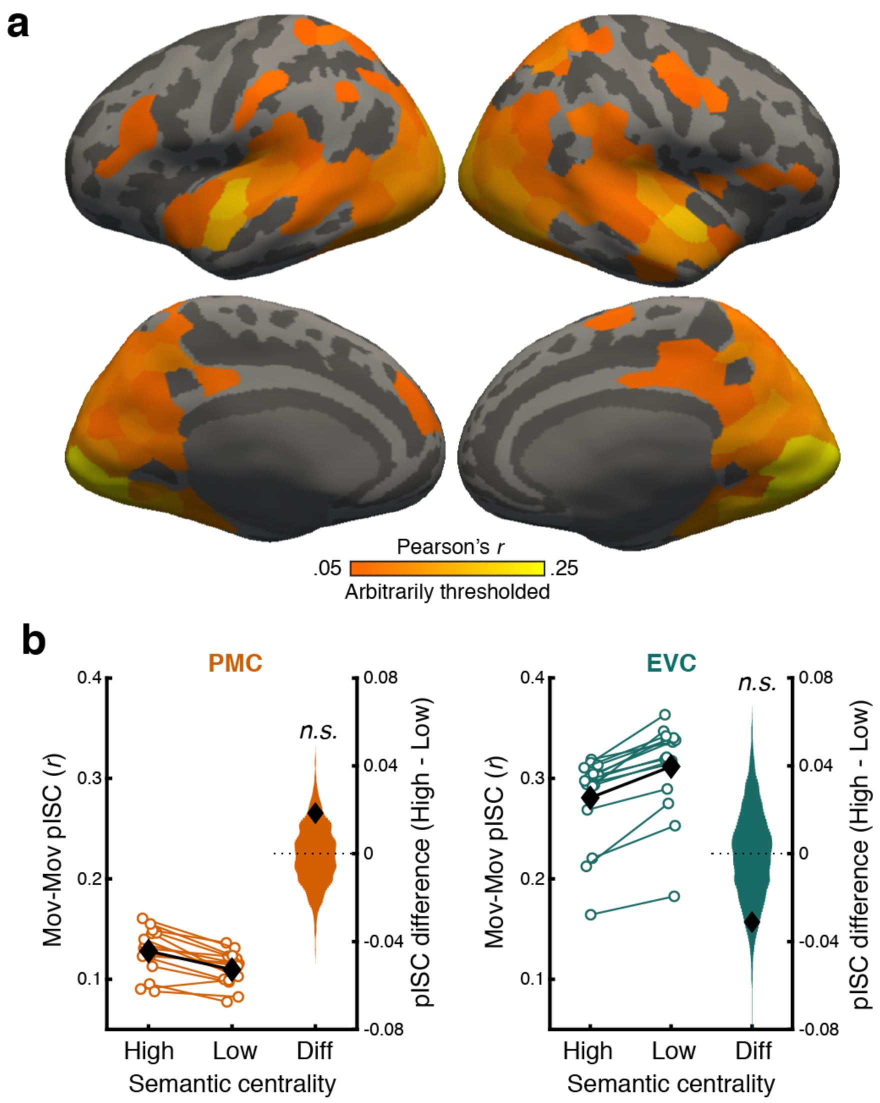

Supplementary Figure 11. Intersubject pattern correlation (pISC) during movie watching. **a.** Whole-brain surface map of mean pISC across matching events during movie watching. The pISC map was arbitrarily thresholded at r = .05 for visualization purposes. pISC values in visualized parcels were all significantly greater than zero (FDR-corrected q < .05 across parcels, one-tailed). **b.** pISC for High vs. Low semantic centrality events during movie watching and the difference (Diff) between the two conditions in the posterior medial cortex (PMC; left) and early visual cortex (EVC; right). For High and Low semantic centrality conditions, white circles represent individual participants (N = 15). Black diamonds represent the mean across participants within each condition. Error bars show SEM across participants. For the difference between High and Low conditions (Diff), black diamonds show the true participant average, and histograms show the null distribution of the mean difference. The difference between High vs. Low semantic centrality events was not significantly different from the null distribution in either ROI (ps > .05, two-tailed randomization tests). Source data are provided as a Source Data file.

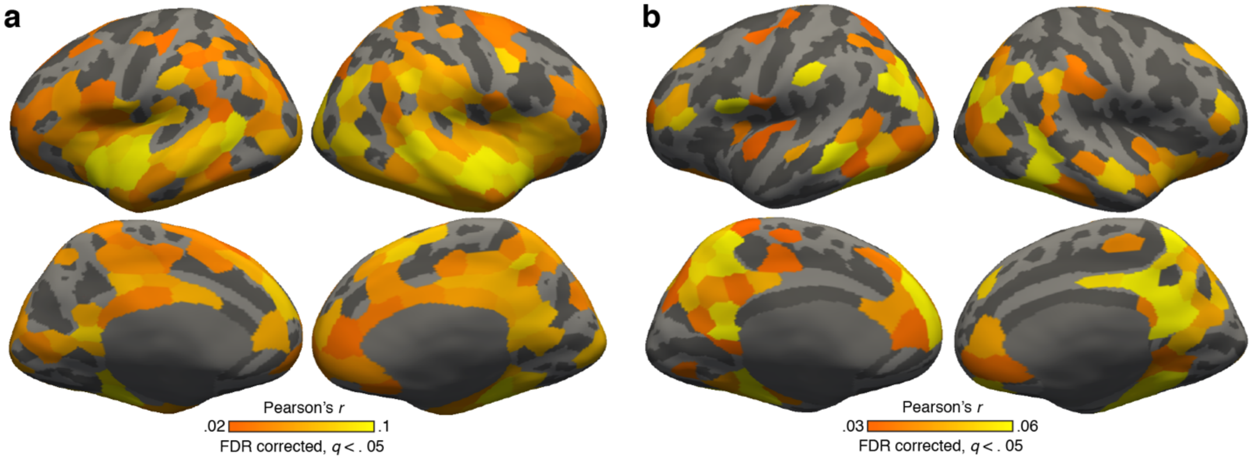

Supplementary Figure 12. Representational similarity analysis using movie watching phase data and recall transcripts. a. Brain regions that show positive correlations between the movie watching phase cross-event intersubject pattern similarity matrix and the movie annotation sentence embedding vector similarity matrix. **b.** Brain regions that show positive correlations between the recall phase cross-event intersubject pattern similarity matrix and the recall transcript sentence embedding vector similarity matrix. The recall transcript similarity matrix was first generated within each participant by computing the cosine similarity between the USE vectors of the participant's recall of movie events. The participant-specific similarity matrices were then averaged across participants. In both **a** and **b**, representational similarity (i.e., fMRI–text correlation averaged across movies and participants) for each parcel was tested for statistical significance against zero using a randomization test (one-tailed). All maps were thresholded at $q < .05$ (FDR-corrected across parcels).

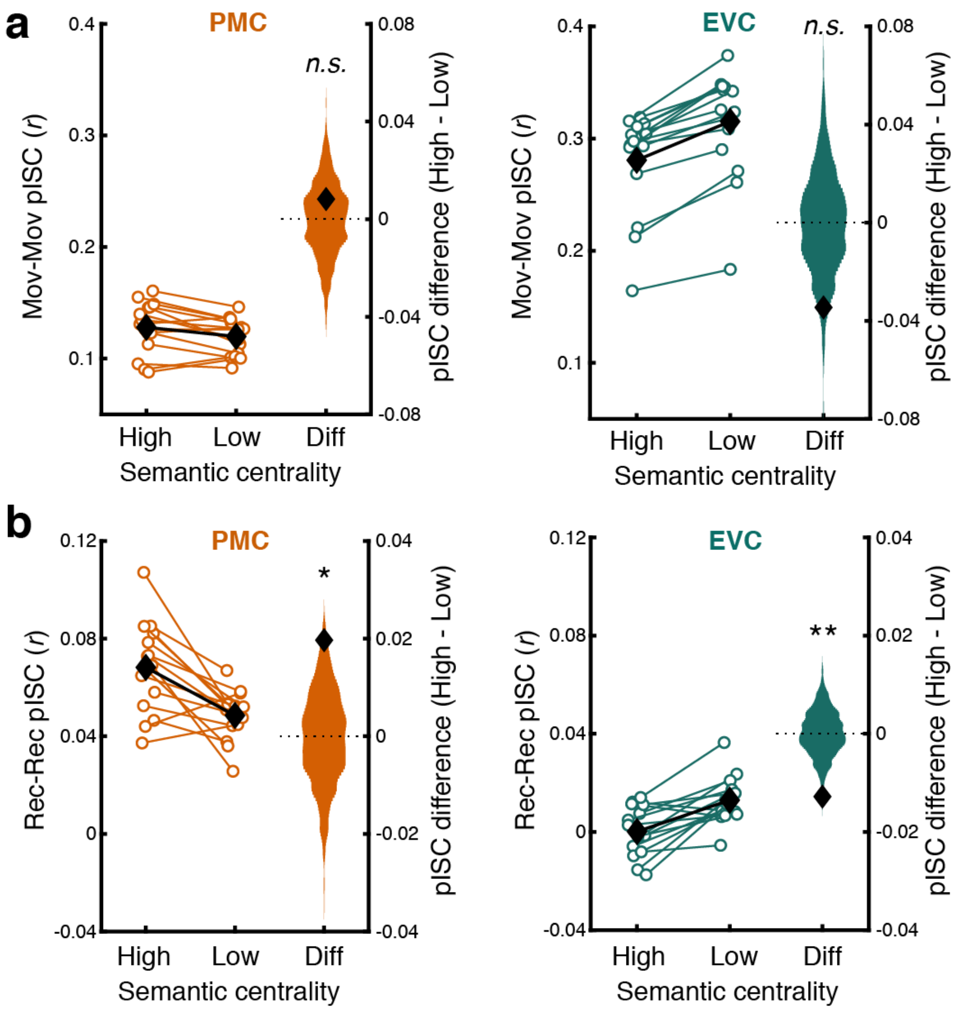

Supplementary Figure 13. Effects of semantic centrality on event-specific intersubject pattern correlation including all events. a & b. Intersubject pattern correlation (pISC) for High vs. Low semantic centrality events and the difference (Diff) between the two conditions during movie watching (**a**) and recall (**b**) in the posterior medial cortex (PMC; left panels) and early visual cortex (EVC; right panels). All movie events were included in the analysis. For High and Low semantic centrality conditions, white circles represent individual participants (*N* = 15). Black diamonds represent the mean across participants within each condition. Error bars show SEM across participants. For the difference between High and Low conditions (Diff), black diamonds show the true participant average, and histograms show the null distribution of the mean difference. Two-tailed randomization tests were performed to test whether the differences between High vs. Low semantic centrality conditions were significantly different from zero. Higher semantic centrality was associated with higher pISC in PMC (p = .039) but lower pISC in EVC (p = .008) during recall. No significant relationship between semantic centrality and pISC was observed during movie watching (ps > .05). Thus, the results were qualitatively identical to those obtained after excluding the first events from movie watching data and after excluding the events recalled by fewer than five participants from recall data. *p < .05, **p < .01. Source data are provided as a Source Data file.

Supplementary References

1. Zou, G. Y. Toward using confidence intervals to compare correlations. *Psychol. Methods* **12**, 399–413 (2007).
2. Cer, D. *et al.* Universal Sentence Encoder. *ArXiv180311175 Cs* (2018).
3. Heusser, A. C., Fitzpatrick, P. C. & Manning, J. R. Geometric models reveal behavioural and neural signatures of transforming experiences into memories. *Nat. Hum. Behav.* **5**, 905–919 (2021).
