# Supplementary material for: Predicting memory from the network structure of naturalistic events
Source: Nat Commun. 2022 Jul 22;13:4235. doi: 10.1038/s41467-022-31965-2 (PMC9307577; doi:10.1038/s41467-022-31965-2)
Supplement: Supplementary file 3 — Reporting Summary [file 41467_2022_31965_MOESM3_ESM.pdf]

nature research

Corresponding author(s): Hongmi Lee
Last updated by author(s): Apr 8, 2022

Reporting Summary

Nature Research wishes to improve the reproducibility of the work that we publish. This form provides structure for consistency and transparency in reporting. For further information on Nature Research policies, see our Editorial Policies and the Editorial Policy Checklist.

Statistics

For all statistical analyses, confirm that the following items are present in the figure legend, table legend, main text, or Methods section.

n/a	Confirmed	
☐	☒	The exact sample size (n) for each experimental group/condition, given as a discrete number and unit of measurement
☐	☒	A statement on whether measurements were taken from distinct samples or whether the same sample was measured repeatedly
☐	☒	The statistical test(s) used AND whether they are one- or two-sided<br>*Only common tests should be described solely by name; describe more complex techniques in the Methods section.*
☐	☒	A description of all covariates tested
☐	☒	A description of any assumptions or corrections, such as tests of normality and adjustment for multiple comparisons
☐	☒	A full description of the statistical parameters including central tendency (e.g. means) or other basic estimates (e.g. regression coefficient) AND variation (e.g. standard deviation) or associated estimates of uncertainty (e.g. confidence intervals)
☐	☒	For null hypothesis testing, the test statistic (e.g. F, t, r) with confidence intervals, effect sizes, degrees of freedom and P value noted<br>*Give P values as exact values whenever suitable.*
☐	☒	For Bayesian analysis, information on the choice of priors and Markov chain Monte Carlo settings
☒	☐	For hierarchical and complex designs, identification of the appropriate level for tests and full reporting of outcomes
☐	☒	Estimates of effect sizes (e.g. Cohen's d, Pearson's r), indicating how they were calculated

Our web collection on statistics for biologists contains articles on many of the points above.

Software and code

Policy information about availability of computer code

Data collection	We used MATLAB (version 2015b) and Psychtoolbox 3 (http://psychtoolbox.org/) for stimulus presentation and response collection during the fMRI experiment. For the pre-registered online experiment, we used Javascript and psiTurk (https://psiturk.org/).
Data analysis	For sentence embedding generation, we used Python 2 and Tensorflow (https://www.tensorflow.org/). For mixed-effects modeling analysis, we used the lme4 package (https://cran.r-project.org/web/packages/lme4/index.html) in R (version 3.4.1). For mediation analysis, we used the mediation package (https://cran.r-project.org/web/packages/mediation/vignettes/mediation.pdf) in R (version 4.0.2). For MRI data preprocessing, we used FreeSurfer 6.0 (recon-all, fsfast), FSL 5.0.10 (MCFLIRT, FUGUE), and custom bash and MATLAB (version 2018b) scripts. For all other analyses of neuroimaging and behavioral data, we used custom MATLAB scripts.

For manuscripts utilizing custom algorithms or software that are central to the research but not yet described in published literature, software must be made available to editors and reviewers. We strongly encourage code deposition in a community repository (e.g. GitHub). See the Nature Research guidelines for submitting code & software for further information.

Data

Policy information about availability of data

All manuscripts must include a data availability statement. This statement should provide the following information, where applicable:

- Accession codes, unique identifiers, or web links for publicly available datasets
- A list of figures that have associated raw data
- A description of any restrictions on data availability

Source data associated with the figures are provided with this paper. The raw fMRI and behavioral data generated in this study have been deposited in OpenNeuro.org (https://doi.org/10.18112/openneuro.ds004042.v1.0.0). The region-of-interest labels, activation maps from univariate analysis, movie annotations, and raw behavioural data from the preregistered online experiment are available at GitHub (https://github.com/jchenlab-jhu/filmfest).

Field-specific reporting

Please select the one below that is the best fit for your research. If you are not sure, read the appropriate sections before making your selection.

☒ Life sciences ☐ Behavioural & social sciences ☐ Ecological, evolutionary & environmental sciences

For a reference copy of the document with all sections, see nature.com/documents/nr-reporting-summary-flat.pdf

Life sciences study design

All studies must disclose on these points even when the disclosure is negative.

Sample size	We recruited 21 participants for the fMRI experiment based on our prior study that showed robust event-specific neural patterns in the default mode network areas (22 participants recruited for Chen et al., 2017, Nature Neuroscience). For the pre-registered online experiment, we recruited 492 participants with the goal of collecting data from approximately 38 participants per movie stimulus after data exclusion. This decision was based on the power analysis (alpha = .05, power = .8) using the effect size (the effect of high vs. low semantic centrality on recall probability) obtained from the behavioral data of the fMRI experiment.
Data exclusions	We excluded 6 participants from the fMRI experiment due to excessive head motion (absolute displacement greater than 4 mm) in at least one scanning run. For the pre-registered online experiment, we excluded 99 participants whose written recall was shorter than 150 words and/or who had watched the movie stimulus before the experiment.
Replication	We conducted one independent pre-registered online experiment to replicate the behavioral effects of semantic and causal centrality found in the fMRI experiment. All behavioral effects were successfully replicated.
Randomization	Randomization was not necessary for the fMRI experiment, as there was only one condition in the experiment (i.e., all participants watched the same movie stimuli and performed the same recall task). For the pre-registered online experiment, each participant was randomly assigned to watch one of 10 movie stimuli based on the date/time they participated in the experiment via Amazon's Mechanical Turk.
Blinding	Blinding was not necessary because our procedures did not involve explicit experimental manipulation.

Reporting for specific materials, systems and methods

We require information from authors about some types of materials, experimental systems and methods used in many studies. Here, indicate whether each material, system or method listed is relevant to your study. If you are not sure if a list item applies to your research, read the appropriate section before selecting a response.

Materials & experimental systems

n/a	Involved in the study	
☒	☐	Antibodies
☒	☐	Eukaryotic cell lines
☒	☐	Palaeontology and archaeology
☒	☐	Animals and other organisms
☐	☒	Human research participants
☒	☐	Clinical data
☒	☐	Dual use research of concern

Methods

n/a	Involved in the study	
☒	☐	ChIP-seq
☒	☐	Flow cytometry
☐	☒	MRI-based neuroimaging

Human research participants

Policy information about studies involving human research participants

Population characteristics	The participants of the fMRI experiment were aged between 20 and 33 years (mean age 26.6 years). There were 12 females and 9 males. All fMRI participants were right-handed native English speakers and reported normal hearing and normal or corrected-to-normal vision. In the online experiment, participants were aged between 18 and 71 years (mean age 38.2), excluding two participants who failed to report their ages. There were 194 females, 198 males, and 1 other gender.
Recruitment	fMRI participants were recruited from the Princeton community. fMRI participants received $20 per hour. Online experiment participants were recruited via Amazon's Mechanical Turk. Online participants received $10 per hour. In the online experiment, additional 99 subjects who had watched the movie stimulus before the experiment or whose written recall was shorter than 150 words were excluded from analyses, leaving subjects who were willing to produce relatively long recall. However, this is unlikely to interact with the effects of event centrality, as subjects were always free to recall any events (regardless of the centrality) in as much detail as they wanted.
Ethics oversight	The fMRI experiment was conducted in accordance with the protocols approved by the Princeton University Institutional Review Board. The pre-registered online experiment was conducted in accordance with the protocols approved by the Johns Hopkins University Institutional Review Board. Informed consent was obtained by participants for both fMRI and online experiments.

Note that full information on the approval of the study protocol must also be provided in the manuscript.

Magnetic resonance imaging

Experimental design

Design type	We used a naturalistic design where participants watched a series of movies and then verbally recalled the movie plots without any explicit constraints.
Design specifications	The movie watching phase of the fMRI experiment consisted of two consecutive scanning runs. Participants watched five movies in each run (first run video duration = 24.9 minutes, second run video duration = 22.9 minutes). The free spoken recall phase immediately followed the movie watching phase. Participants were instructed to describe aloud what they remembered from the movies in as much detail as they could, regardless of the order of presentation. Participants verbally indicated that they were finished by saying “I'm done” after recalling everything they could remember. In case participants needed to take a break or the duration of the scanning run exceeded the scanner limit (35 minutes), we stopped the scan in the middle and started a new scanning run where the participants resumed from where they had stopped in the previous run.
Behavioral performance measures	We recorded participants' spoken free recall as audio files.

Acquisition

Imaging type(s)	functional and structural
Field strength	3 T
Sequence & imaging parameters	Functional images were acquired using a T2*-weighted multiband accelerated echo-planar imaging (EPI) sequence (TR = 1.5 s; TE = 39 ms; flip angle = 50°; acceleration factor = 4; shift = 3; 60 oblique axial slices; grid size 96 × 96; voxel size 2 × 2 × 2 mm3). Fieldmap images were also acquired to correct for B0 magnetic field inhomogeneity (60 oblique axial slices; grid size 64 × 64; voxel size 3 × 3 × 2 mm3). Whole-brain high-resolution anatomical images were acquired using a T1-weighted MPRAGE pulse sequence. Scanning parameters for the anatomical images varied across subjects (15 subjects had 176 sagittal slices with voxel size 1 × 1 × 1 mm3; 6 subjects had 192 sagittal slices with voxel size .9 × .86 × .86 mm3), as the anatomical images of a subset of subjects were originally obtained for other projects unrelated to the current study.
Area of acquisition	whole brain

Diffusion MRI ☐ Used ☒ Not used

Preprocessing

Preprocessing software	We used FreeSurfer's recon-all pipeline (version 6.0) and FSL 5.0.10 for the preprocessing of functional and structural images. Functional images were corrected for motion and B0 magnetic inhomogeneity, coregistered to the structural images, resampled to the template brains, smoothed (FWHM 4mm), and high-pass filtered (cutoff = 140 s).
Normalization	For volume analysis, functional images were normalized using linear transformation. For surface analysis, spherical registration involving nonlinear transformation was performed.
Normalization template	Functional images were normalized to the MNI 305 volume space (for subcortical/volume analysis) and the fsaverage6 template surface (for cortical/surface analysis).

Noise and artifact removal

Functional images were corrected for head motion using the first volume of each run as the reference volume (degree of freedom = 6). For intersubject functional connectivity analysis, we additionally projected out the following nuisance regressors: the average time courses (z-scored within each run) of 1) high s.d. voxels outside the grey matter mask (voxels in the top 1% largest s.d.), 2) cerebrospinal fluid, and 3) white matter.

Volume censoring

Volume censoring was not applied.

Statistical modeling & inference

Model type and settings

For whole-brain univariate activation analysis, we first computed the mean activation for each movie event by averaging the preprocessed BOLD signal across TRs that correspond to the event. We then performed a linear regression where the event-by-event activation (combined across all 10 movies) was explained by the semantic or causal centrality of the events, after regressing out the overall movie-level activation from the event-by-event activation. Finally, one-sample t-tests against zero were applied on the participant-specific vertex-wise parameter estimate maps to generate the group-level t-statistic map.

Intersubject pattern correlation (pISC) analysis was performed as follows: for each brain region of each participant, first the mean activation pattern of each event was generated by averaging the preprocessed BOLD data across TRs within the event in each vertex within the region. For each participant and event, we computed the Pearson correlation between the event pattern of the participant and the pattern of the matching event from each of the remaining participants, which resulted in N – 1 correlation coefficients (N = the total number of participants who watched or recalled the event). The correlation coefficients were then averaged to create a single pISC (r) value per event per participant. These pISC values were averaged across events (combined across all 10 movies) and participants, resulting in a single pISC value for each region.

Representational similarity analysis (RSA; Kriegeskorte et al., 2008) was performed by comparing event-by-event similarity matrices based on 1) the text descriptions of events and 2) fMRI patterns. To create the fMRI-based similarity matrix for each movie, we computed pattern correlations between all possible pairs of events between all pairs of participants. For each participant and movie, this resulted in N – 1 fMRI pattern similarity matrices, where N is the total number of participants. We took the average of each matrix and its transpose to make the similarity matrix symmetric, and then averaged the N – 1 similarity matrices to generate a single fMRI similarity matrix per movie and participant. The representational similarity between the text-based similarity matrix and the fMRI pattern-based similarity matrix was measured by computing the Pearson correlation between the lower triangles (excluding the diagonal values) of each matrix. The correlation coefficients were next averaged across movies and then across participants to create a single r value per region.

Effect(s) tested

In the whole-brain univariate analysis, we used one-sample t-tests to test whether the semantic or causal centrality of events scaled with BOLD responses (i.e., positive beta estimates for the centrality regressor). We also used paired t-tests to test whether average activation within a region of interest was greater for high than low centrality events. The whole-brain pISC analysis tested whether the average event-specific pISC was positive in each parcel. We also tested whether pISC was greater for high than low centrality events within each region of interest. The whole-brain RSA tested whether there was positive correlation between the event-by-event similarity matrices based on text descriptions (either from movie annotations or recall transcripts) and fMRI activation patterns. We used nonparametric randomization tests to test the statistical significance of pISC and RSA results.

Specify type of analysis: ☐ Whole brain ☐ ROI-based ☒ Both

Anatomical location(s)

Anatomical locations were determined based on a cortical parcellation atlas (Schaefer et al., 2018) and a subcortical atlas (FreeSurfer's Aseg) on standard template brains.

Statistic type for inference
(See Eklund et al. 2016)

For the whole-brain pISC and RSA, parcel-wise statistical inference (nonparametric randomization test) was applied. Vertex-wise statistical inference (one-sample t-test) was applied in the whole-brain univariate activation analysis.

Correction

For the whole-brain pISC and RSA, we applied the Benjamini-Hochberg procedure ($q < .05$) to correct for multiple comparisons across cortical parcels. We also applied the Benjamini-Hochberg procedure ($q < .05$) to correct for multiple comparisons across time points in the hippocampal time series comparison.

Models & analysis

n/a	Involved in the study
☐	☒ Functional and/or effective connectivity
☒	☐ Graph analysis
☒	☐ Multivariate modeling or predictive analysis

Functional and/or effective connectivity

Intersubject functional connectivity (Simony et al., 2016) was computed between the hippocampus and the cortex within each movie event. For each participant, we correlated the participant's hippocampal time series of the event and the cortical ROI time series averaged across all other participants. We then averaged the Pearson correlation coefficients across all participants. This procedure was repeated by correlating each participant's cortical ROI time series and the hippocampal time series averaged across all other participants. Again, the correlation coefficients were averaged across participants. We then took the mean of the two averaged correlations to produce a single ISFC between the hippocampus and the cortical ROI for each event.
